# A study on the relationship between odor hedonic ratings and individual odor detection threshold

**DOI:** 10.1038/s41598-022-23068-1

**Published:** 2022-11-02

**Authors:** Charlotte Bontempi, Laurence Jacquot, Gérard Brand

**Affiliations:** 1 Laboratoire de Recherches Intégratives en Neurosciences et Psychologie Cognitive – UR481, University of Franche-Comté, 25000 Besançon, France; 2 CSGA Centre des Sciences du Gout et de l’AlimentationCSGA Centre des Sciences du Gout et de l’Alimentation, University of Franche-Comté, 21000 Dijon, France

**Keywords:** Olfactory system, Neuroscience, Sensory processing

## Abstract

Odor hedonic perception (pleasant/unpleasant character) is considered as the first and one of the most prominent dimensions in olfaction and is known to depend on several parameters. Among them, the relation between the odorant concentration and the hedonic estimation has been widely studied. However, few studies have considered odor hedonic ratings (OHR) in relation to individual detection thresholds (IDT). Thus, the aim of this study was to determine olfactory detection thresholds and to describe hedonic rating variations from individual thresholds to higher concentrations. IDT were performed for two pleasant (apple and jasmine) and two unpleasant (durian and trimethylamine) odorant stimuli. The experimenter presented one by one in a randomized order, the different odorant concentrations above IDT. Participants rated odor hedonic valence of these stimuli on a visual analog scale. Results showed, except for trimethylamine, the same relationship between hedonic ratings and stimulus concentration, i.e., an increase of pleasantness (apple and jasmine)/unpleasantness (durian) ratings at low and middle concentrations followed by a plateau at high concentrations. Correlations between OHR and concentrations as well as between OHR and threshold steps were always significant. Moreover, comparisons between both conditions showed that the correlation coefficient was significantly higher for trimethylamine (and a trend for apple) when IDTs were considered, while no difference was found for jasmine and durian. Overall, results suggested that the relationship between OHR and IDT is odor specific. These findings contribute to explain the large variability of the hedonic tone (i.e., weakly vs. very pleasant, weakly vs. very unpleasant) at specific concentration in the general population and could serve future research in this field (e.g., olfactory preferences in nutrition studies, anhedonia in psychiatric disorders…).

## Introduction

In humans, the sense of smell is implied in large number of adaptative behaviors in response to olfactory inputs from the environment^1^. Nevertheless, olfactory perception appears as a complex and multidimensional process, partly due to the salient affective dimension of odors. Indeed, odor hedonic perception is the first and the most prominent response following an olfactory stimulation^2^.

In recent years, the study of invariants in odor hedonic perception remained a recurring question. Some studies demonstrated that odor hedonic perception could be predicted by odorant structure^3–5^, and could be related to the molecular complexity of odorants or to the number of olfactory notes^6,7^, and especially to molecule weight and molecular size^8,9^. Yet, several studies showed a great flexibility of odor hedonic perception. Thus, differences were observed in relation to individual characteristics such as age^10–12^, sex^13–18^, the experience towards odorants^19^ and physiological state^20,21^, diseases such as depressive disorders^22^, schizophrenia^23,24^ or Parkinson’s disease^25,26^ and recently with SARS-CoV-2^27^. Odor hedonic perception also appeared to depend on repeated exposures^28,29^, verbal influence^30,31^, or stimulus presentation pathway (orthonasal vs. retronasal)^32^. Contrary to individual differences, it seems that the perception of odor pleasantness is similar across cultures in adults^33^ as well as in children^34^.

In addition, it was demonstrated that the stimulus concentration influenced pleasantness/unpleasantness ratings^13,35–39^. Taken together, these studies suggested that for a pleasant odor, hedonic ratings increased with increasing odorant concentrations. Jointly, for an unpleasant odor, hedonic ratings decreased with increasing odorant concentrations. However, in some above-mentioned studies, other patterns were suggested with an increase of odor hedonic ratings (OHR) (or a decrease for unpleasant odorants) until a plateau for the higher concentrations. For some pleasant odorants, it could even be observed for the highest concentrations, a decrease of hedonic ratings following the plateau.

Surprisingly, no studies have really taken into account individual olfactory detection thresholds (IDT) in odor hedonic evaluation. Indeed, even though some works studied pleasantness and odor detection thresholds^40,41^ the relation between both parameters was never examined. Marginally, it was suggested that odor detection thresholds of subjects who consistently rated some odors as pleasant were higher than those of subjects who rated them as unpleasant, although no evidence was given^13^. Recently, Liu et al.^42^ measured the affective appraisal of 40 odors and odor detection threshold of each participant with a standard “Sniffin’Sticks” test^43^. Authors demonstrated no relationship between odor valence and odor detection threshold^42^. However, Liu et al., measured the odor detection thresholds using the standard “Sniffin’ Sticks” test^43^, corresponding to a detection threshold for a specific odorant (here, PEA). This cannot be generalized to a general sensitivity for a subject insofar as the odor detection thresholds are known to vary from an odorant to another. Furthermore, IDTs present great intra- and inter-individual differences in relation to several factors identified in many studies^44^.

Thus, the aim of the present work was to determine whether the IDT to a specific odorant stimulus influences OHR of this odorant, from the threshold to higher concentrations. Based on previous studies, two pleasant odors (apple and jasmine)^45,46^ and two unpleasant odors (durian and trimethylamine)^11,47,48^ were used in a homogenous population (academic level and age-matched).

## Material and methods

### Participants

Twenty-six volunteer subjects participated in the entire experiment. All participants were undergraduate students from the University of Franche-Comté (France) (8 men, 18 women) and reported normal smell sensitivity, i.e., none of them had a history of nasal/sinus disease, extensive exposure to chemicals with potential toxicity (including cigarette smoke) or long-term medical treatment. Their age ranged from 21 to 26 years (M_age_ = 22.5 ± 1.65 years). The study was conducted in accordance with the Declaration of Helsinki-Hong Kong and the study design was approved by the Human Protection Committee East Area II (Besançon, France). Each participant gave his/her written informed consent prior to inclusion in the study.

### Odor stimuli

Four odorant stimuli were selected on the basis of their hedonic valence (two pleasant and two unpleasant) determined in a preliminary experiment. Pleasant odors were apple (Meilleurduchef^®^, 58 mL, ref: pomm58, France) and jasmine (Colichef^®^, 125 mL, France) and unpleasant odors were durian (a popular tropical fruit in Southeast Asia, smelling like decay^49^) (Culinaide^®^, 105 mL, France) and trimethylamine (having an unpleasant fish smell^50^) (trimethylamine solution ~ 45 wt% in H_2_O, ref: 92262, SigmaAldrich^®^, Germany).

Thirty-five dilutions for apple, jasmine and durian odors were prepared into a 5mL test-tube using a 2-fold serial dilutions method protocol with a final volume of 2mL. Thus, considering a 0.5 factor dilution, the first test-tube (named Concentration 1) contained 50.000% v/v solution and the last one, i.e., the most diluted named Concentration 35, contained a 2.910 × 10^−11^ % v/v solution. The stock solution was named Concentration 0.

For trimethylamine solution, 40µL of the stock solution was first diluted into 3.960 mL of distilled water. This new solution was named Concentration 0. Then, from the Concentration 0, 50 dilutions of trimethylamine were prepared with the same method as for the three other odorant stimuli. Thus, the first test-tube (Concentration 1) contained a 50.000% v/v solution and the last one (the 50th concentration), a 8.882 × 10^−16^ % v/v solution.

According to the serial two-fold dilution used in the present study and because IDTs are known to be particularly low for trimethylamine^51,52^, 50 dilutions were needed for this odorant.

### Procedure

The experiment was carried out in a quiet and well-ventilated room located in the University of Franche-Comté and equipped with an individual booth. Upon arrival, participants gave written informed consent. Then, they were invited to fill out a personal information questionnaire (age, sex, hunger level).

For each odorant condition, the experiment was divided into two parts. The first part consisted in IDT determination and the second part was dedicated to OHR. For each odor, all participants performed the test in four different sessions (i.e., one session by odor) separated by a week.

Participants were asked not to consume any food or drink (except water) at least two hours before the test to minimize the satiety state effect on olfactory tests. Hunger level was assessed using a visual analog scale ranging from 0 (“not hungry at all”) to 10 (“very hungry”) to verify that hunger level was homogenous between participants and between sessions. The experimental room was ventilated 15 minutes prior to participants’ arrival. The experiment was conducted before the SARS-CoV-2 pandemic.

### Detection thresholds

Participants were blindfolded to prevent visual identification of the test-tube containing the odorant stimulus. Detection thresholds for apple, jasmine, durian, and trimethylamine odors were performed from the concentration ranges described above. The method used was based on the Sniffin’Sticks test developed by Hummel et al. (1997), using a single staircase method in a triple-forced-choice paradigm. Three test tubes were presented to each participant in a randomized order: two contained distilled water and the other the odorant at a particular concentration. The subject had to indicate the test-tube containing the odorant. Odorant concentrations were presented in ascending order until participants had correctly discerned the odorant in two successive trials. Then, odorant concentrations were presented in descending order until participants had incorrectly discerned the odorant, and so on. The last four staircase reversal points were used to estimate the IDT. Once the IDT was determined, the experimenter listed the concentrations above the subject's detection threshold for the given odor. Concentrations above the IDT of the participant, in step of 3 (threshold, threshold + 3, threshold + 6...), were selected before creating a random presentation order for the following part of experiment, i.e., the odor hedonic ratings. This part lasted from 15 to 25 min.

### Odor hedonic ratings

Participants sat on a chair in an individual booth facing a screen computer. OHRs were performed with FIZZ Biosystems (Biosystèmes, Couternon, France), a sensory analysis software allowing automated data collection. For each odorant concentration, hedonic rating was evaluated using a visual analog scale from − 9 to + 9 (“strongly unpleasant” to “strongly pleasant”). The experimenter presented one by one in a randomized order, the different odorant concentrations selected at the end of the first part (i.e., IDT) including the concentration 0. The number of odor presentations varied between subjects according to the individual thresholds (apple: mean ± SEM = 8.46 ± 0.51; jasmine: mean ± SEM =7.69 ± 0.40; durian: mean ± SEM = 7.50 ± 0.47; trimethylamine: mean ± SEM = 11.21 ± 0.82).

The odorant at a specific concentration was inhaled only one time by participants before scale completing. The time interval between each concentration corresponded to the time required to complete the odor hedonic scale. During the test, participants were not aware of the name of the odor used and that the different concentrations presented corresponded to the same odorant. Odor presentation order was randomized between participants. This part lasted from 10 to 15 min.

### Data analysis

Data for IDTs and OHRs were statistically evaluated with Statistica software^®^, using Spearman correlations, Wilcoxon tests, Mann-Whitney U tests, Fisher Z-transformation and polynomial regressions. The level of significance was *p* < 0.05.

### Ethics approval and informed consent

The present study was conducted in accordance with the Declaration of Helsinki-Hong Kong. This study design was approved by the Human Protection Committee East Area II (Besançon, France). Each participant gave his written informed consent prior to inclusion in this study.

## Results

### Odor detection thresholds

**Figure 1:**
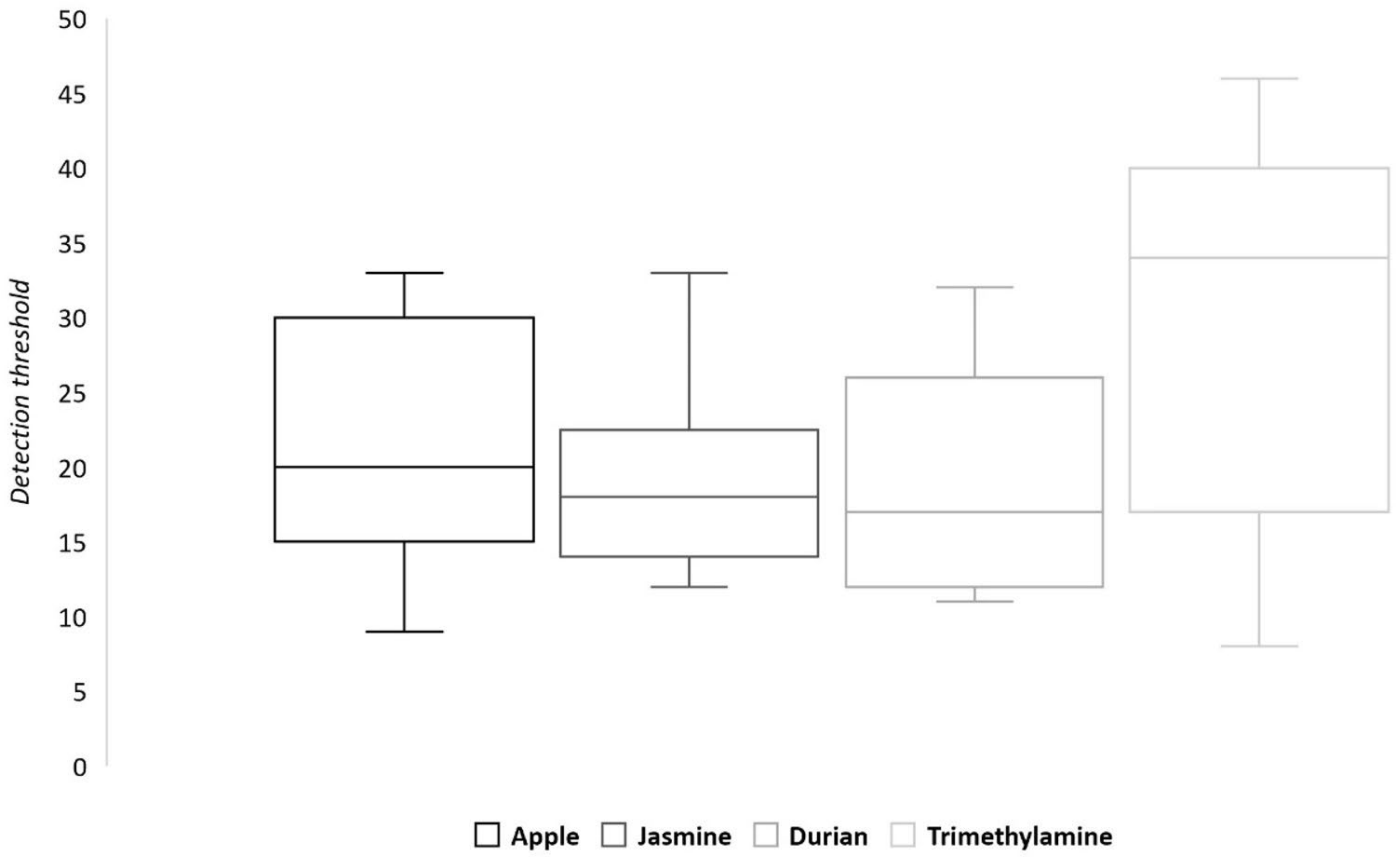
Boxplots of the IDT for apple, jasmine, durian, and trimethylamine odorants. In each boxplot, the middle line of the box corresponds the mean of odor detection threshold, the inferior line of the box corresponds to the first quartile, and the upper line of the box corresponds to the third quartile.

Results are reported in Fig. 1.

For apple and jasmine, IDTs ranged from concentrations 9 to 33, and 12 to 33 respectively. A majority of participants had an IDT comprised between concentrations 15 and 30 for apple and between concentrations 14 and 22 for jasmine.

Concerning durian, IDTs ranged from concentrations 11 to 32 and most of participants had a IDT comprised between concentrations 12 and 26. For trimethylamine odor, the dispersion of detection thresholds appeared wider than for durian (from concentration 8 to concentration 46) with a majority of participants reaching IDT between concentrations 18 and 40.

Spearman correlations were performed between the four odorants (Table 1). Results showed no significant results except a positive correlation between IDTs for apple and jasmine (*ρ* = 0.448, *p* < 0.05).

**Table 1:** Spearman correlations between IDT.

	Spearman *ρ*	*p* value
Apple/Jasmine	0.448	*p* < 0.05
Durian/Trimethylamine	0.207	NS
Apple/Durian	0.105	NS
Apple/Trimethylamine	− 0.233	NS
Jasmine/Durian	0.354	NS
Jasmine/Trimethylamine	0.047	NS

Significant results were considered for *p* < 0.05. Non-significant results are noted as NS.

### Hedonic ratings at detection thresholds

**Figure 2:**
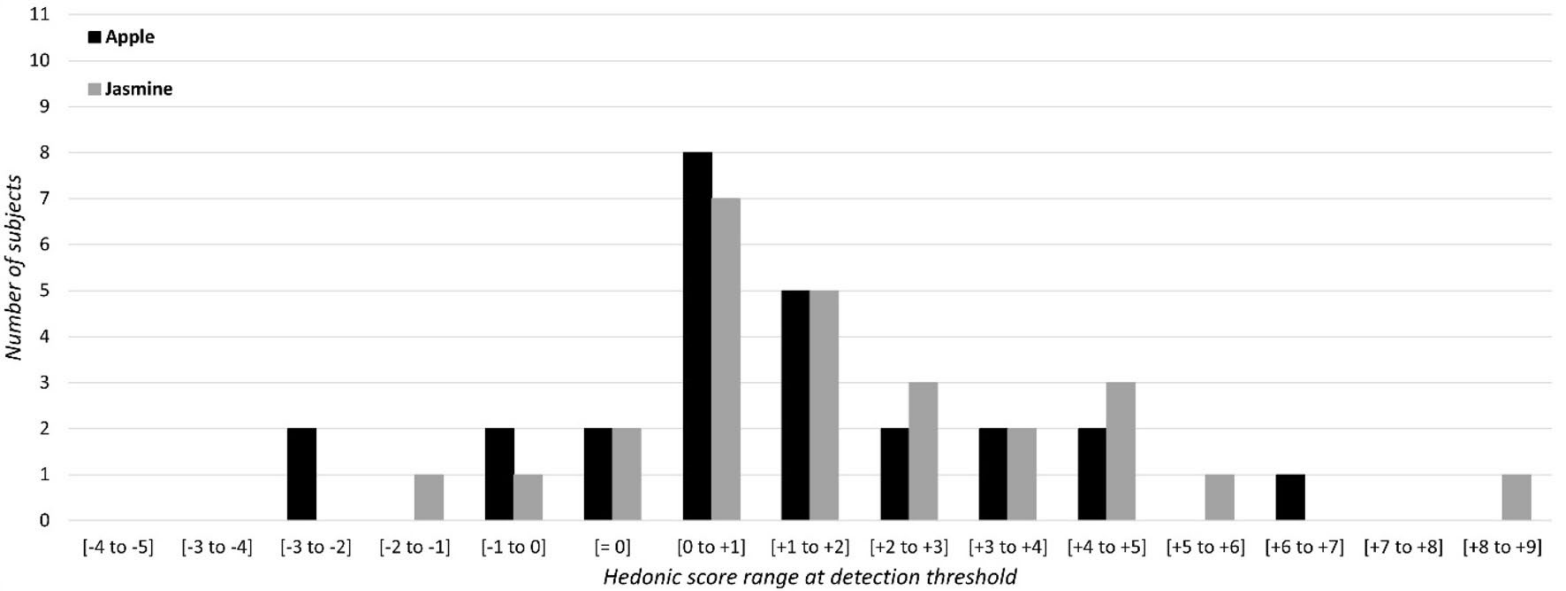
Hedonic scores at IDT for pleasant odors: number of subjects for each score range (e.g., [− 2 to − 1] means that odor hedonic rating is comprised between − 2 and − 1). OHR were assessed on a visual analog scale ranging from 0 to + 9 for the pleasant polarity and from 0 to − 9 for the unpleasant polarity.

**Figure 3:**
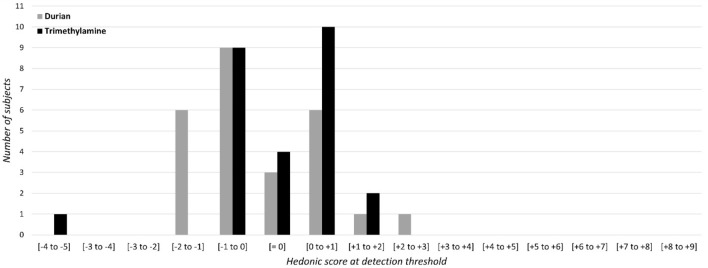
Hedonic scores at IDT for unpleasant odors: number of subjects for each score range (e.g., [− 2 to − 1] means that OHR is comprised between − 2 and − 1). OHR were assessed on a visual analog scale ranging from 0 to + 9 for the pleasant polarity and from 0 to − 9 for the unpleasant polarity.

Results for pleasant odors are reported in Fig. 2. The distribution of hedonic scores in relation to IDT appears similar for both apple and jasmine odors. Most of the subjects rated apple and jasmine odors at their threshold to being very slightly pleasant (hedonic scores ranging from 0 and + 2). However, one can note a dispersion along the hedonic scale with some subjects rating the odors as slightly unpleasant at their detection threshold. Results for unpleasant odors are reported in Fig. 3. The distribution of hedonic scores in relation to IDT appears mainly aggregated around the 0 score, ranging from − 2 to + 2 for durian and from − 1 to + 2 for trimethylamine. Several subjects rated the odor as slightly pleasant at their detection threshold.

A Wilcoxon test was performed to compare OHR at IDT for (1) pleasant odors and (2) unpleasant odors. Results showed no significant difference neither between apple and jasmine odors (Z= 0.73, *p* = 0.45) nor between durian and trimethylamine (Z= 1.01, *p* = 0.31). A Wilcoxon test was thereby performed to compare OHR at IDT between odors groups, i.e., between the pleasant odors group (apple + jasmine) and the unpleasant odors group (durian + trimethylamine). Results indicated a clear difference of hedonic ratings at IDT between odors groups, suggesting that unpleasant odors were rated as significantly more unpleasant than pleasant odors at IDT (Z= 5.36, *p* < 0.001) (Table 2).

**Table 2:** Wilcoxon test for dependent samples of OHR at detection threshold between apple and jasmine, between trimethylamine and durian, and between odor groups (pleasant odors with apple + jasmine and unpleasant odors with durian + trimethylamine).

	Z	*p* value
Apple/Jasmine	0.73	NS
Trimethylamine/Durian	1.01	NS
Pleasant/Unpleasant odors	5.36	*p* < 0.001

Significant results were considered for *p* < 0.05. Non-significant results are noted as NS.

Spearman correlations were performed between the four odorants. Only one significant correlation was observed between durian and trimethylamine hedonic scores at IDT (*ρ* = 0.459, *p* < 0.02) (Table 3).

**Table 3:** Spearman correlations between OHR at IDT.

	Spearman *ρ*	*p* value
Apple/Jasmine	0.110	NS
Durian/Trimethylamine	0.459	*p* < 0.02
Apple/Durian	0.359	NS
Apple/Trimethylamine	− 0.08	NS
Jasmine/Durian	− 0.104	NS
Jasmine/Trimethylamine	0.009	NS

Significant results were considered for *p* < 0.05. Non-significant results are noted as NS.

### Hedonic ratings above odor detection thresholds

#### Analysis as a function of concentrations

**Figure 4:**
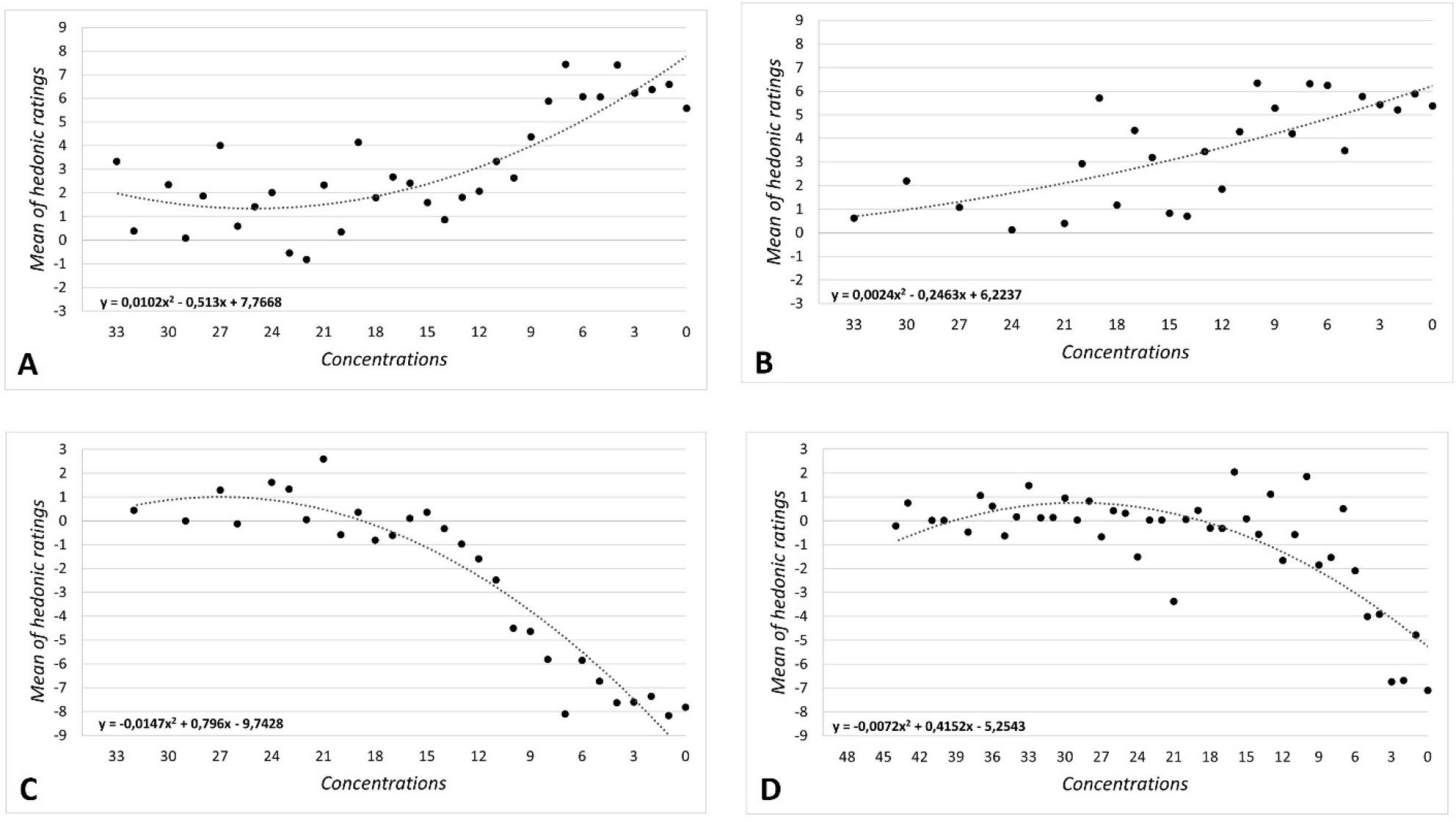
Odor hedonic ratings above odor detection thresholds: mean hedonic ratings in relation to concentrations. (**A**) apple, (**B**) jasmine, (**C**) durian, (**D**) trimethylamine. The line grey color responds to the polynomial regression. Equations of polynomial regressions is given on each graph.

Mean hedonic scores obtained for each concentration and each odor used are reported in Fig. 4, including the equations of the polynomial regressions.

For pleasant odors, the analysis showed a positive polynomial regression. Thus, when the concentration increased, mean hedonic scores increased (from neutral to very pleasant). For apple odor, hedonic scores from concentration 33 to concentration 11 were rather stagnant. This was followed by an increase of odor hedonic scores from concentration 9 to concentration 0 (i.e., pure solution). For jasmine odor, a less pronounced tendency was observed, with rather stagnant hedonic scores from concentration 33 to concentration 21, followed by a steady increase.

For unpleasant odors, the analysis indicated a negative polynomial regression. Thus, when the concentration increased, mean hedonic scores decreased (from neutral to very unpleasant). As for pleasant odors, results showed a similar phenomenon of stagnant hedonic scores for the lower concentrations. For durian, from concentration 33 to 21, mean hedonic scores were rather stagnant. This was followed by a decrease of odor hedonic scores from concentration 20 to concentration 0 (i.e., pure solution), suggesting that durian odor was perceived as more unpleasant. For trimethylamine, mean hedonic scores were rather stagnant from concentration 45 to 15 (ranging between − 1 and + 1). This was followed by a decrease from concentration 14 to concentration 0 (i.e., pure solution) meaning that trimethylamine odor was perceived as more unpleasant.

#### Analysis as a function of individual detection thresholds

**Figure 5:**
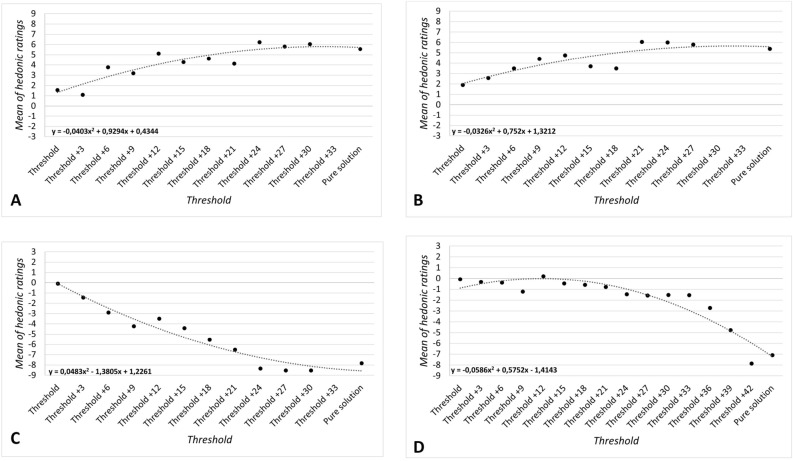
Odor hedonic ratings above odor detection thresholds: mean hedonic ratings in relation to individual detection threshold in step of 3 concentrations. (**A**) apple, (**B**) jasmine, (**C**) durian, (**D**) trimethylamine. The line grey color responds to the polynomial regression. Equations of polynomial regressions is given on each graph.

Mean hedonic scores obtained for concentrations above threshold, in step of 3, are reported in Fig. 5, including the equations of the polynomial regressions.

For both pleasant odors, the polynomial regression curves were strongly similar with an increase of the hedonic scores, from + 2 (threshold + 3) to + 6 (threshold + 33). On the other hand, for both unpleasant odors the polynomial regression showed a different pattern. For durian, results showed a decrease of hedonic scores from threshold to threshold + 21, which is followed by a plateau (with, in this case a ceiling effect). For trimethylamine data demonstrated a plateau from threshold to threshold + 33, followed by a decrease of hedonic scores.

#### Correlations

Spearman correlations were performed (1) between OHR and concentrations (i.e., analysis independent of IDT) and (2) between OHR and threshold step (i.e., analysis considering IDT). Correlation coefficients and *p* values are displayed in Table 4.

**Table 4:** Spearman correlations between OHR and concentrations, and between OHR and threshold step for each odor session.

	Relation between OHR and concentrations	Relation between OHR and threshold step	Z comparisons
Apple	*ρ* = − 0.702, *p* < 0.001 Z_Fisher_ = − 0.87	*ρ* = 0.869, *p* < 0.001 Z_Fisher_ = 1.29	1.55, NS
Jasmine	*ρ* = − 0.718, *p* < 0.001 Z_Fisher_ = − 0.708	*ρ* = 0.790, *p* < 0.01 Z_Fisher_ = 1.07	0.57, NS
Durian	*ρ* = 0.903, *p* < 0.001 Z_Fisher_ = 1.48	*ρ* = − 0.949, *p* < 0.001 Z_Fisher_ = − 1.82	1.13, NS
Trimethylamine	*ρ* = 0.508, *p* < 0.001 Z_Fisher_ = − 1.48	*ρ* = − 0.932, *p* < 0.001 Z_Fisher_ = 2.04	3.77, *p* < 0.05

Significant results were considered for *p* < 0.05. It must be noted that the negative correlations values are due to the reverse coding of concentration levels (lower number = higher concentration).

Results showed significant correlations between OHR and concentrations for all odors (apple: *ρ* = − 0.702, *p* < 0.001; jasmine: *ρ* = − 0.718, *p* < 0.001: durian: *ρ* = 0.903, *p* < 0.001: trimethylamine: *ρ* = 0.508, *p* < 0.001). Similar data were obtained for the correlation analysis between OHR and threshold step (apple: *ρ* = 0.869, *p* < 0.001; jasmine: *ρ* = 0.790, *p* < 0.01; durian: *ρ* = − 0.949, *p* < 0.001; trimethylamine: *ρ* = − 0.932, *p* < 0.001) (Table 4).

A Fisher Z-transformation was applied to each correlation coefficient to perform statistical comparisons (Table 4). Results demonstrated that, when IDT was taken into account, correlation coefficient was significantly higher for trimethylamine. Besides, a trend towards significance was also shown for apple, while no difference was found for jasmine and durian.

### Sex and hunger level effect

Concerning sex effect, a Mann-Whitney U test was conducted between men and women to compare IDT as well as OHR at IDT and pure solution for the four odorants used. Data are presented in Table 5 and revealed no sex effect on IDT and OHR for all odorants used.

**Table 5:** Mann–Whitney U tests: sex effect on IDT, OHR at detection threshold and at pure solution for all odors used.

	IDT	OHR at detection threshold	OHR at pure solution
Apple	U = 39.5 NS	U = 55.0 NS	U = 61.0 NS
Jasmine	U = 46.0 NS	U = 57.0 NS	U = 62.5 NS
Durian	U = 57.0 NS	U = 64.0 NS	U = 44.0 NS
Trimethylamine	U = 62.5 NS	U = 57.5 NS	U = 51.0 NS

Non-significant results are noted as NS.

Mean (and SEM) hunger level of participants in each odor session are presented in Table 6. Results of Wilcoxon tests showed no significant difference between the different odor sessions (Table 7). Moreover, results of Spearman rank correlations did not show any significant correlation between hunger level and IDT, OHR at detection threshold and at pure solution whatever the odor session (Table 8).

**Table 6:** Mean and SEM of participants’ hunger level in each odor session.

	Apple	Jasmine	Durian	Trimethylamine
Mean	4.54	4.62	4.85	5.7
SEM	0.67	0.68	0.57	0.63

Hunger level was evaluated using a visual analog scale ranging from 0 (“not hungry at all”) to 10 (“very hungry”).

**Table 7:** Wilcoxon tests: comparisons of hunger level between the different odor sessions.

Odor session	Z	*p* value
Apple/Jasmin	0.08	NS
Apple /Durian	0.44	NS
Apple/Trimethylamine	1.13	NS
Jasmine/Durian	0.63	NS
Jasmine/Trimethylamine	1.51	NS
Durian/Trimethylamine	0.72	NS

Non-significant results are noted as NS.

**Table 8:** Spearman rank correlations between hunger level and IDT, OHR at detection threshold/at pure solution in each odor session. Non-significant results are noted as NS.

Odors	IDT	OHR at detection threshold	OHR at pure solution
Apple	ρ = − 0.266 NS	ρ = − 0.231 NS	ρ = − 0.330 NS
Jasmine	ρ = 0.007 NS	ρ = 0.02 NS	ρ = 0.04 NS
Durian	ρ = − 0.005 NS	ρ = − 0.122 NS	ρ = − 0.08 NS
Trimethylamine	ρ = 0.180 NS	ρ = 0.08 NS	ρ = 0.07 NS

## Discussion

First, these findings confirmed that odor IDT showed great interindividual variability, even in an age-homogeneous population^44^. This is in accordance with previous studies demonstrating that variability in odor detection thresholds occurs within individuals across time^53,54^. In the present study, with a 0.5 dilution factor, results indicated a difference of thirty concentrations between two subjects, which corresponds to a ten billion vol/vol ratio. Moreover, the detection threshold for a specific odorant was a weak indicator of the detection threshold for another odorant insofar as no correlation has been found between the four odorants, except between apple and jasmine. Thus, the use of a single concentration to test hedonic ratings in a large population raises several problems: some subjects with a high threshold (i.e., a low sensitivity) could not perceive the odorant stimulation and the hedonic scores could be dependent on the individual thresholds.

Second, data relating to hedonic scores at the IDT showed that most subjects rated the pleasantness as weakly pleasant or weakly unpleasant. It can be hypothesized that at detection threshold, the perception of the stimulus is weak, and the subjects are unable to recognize the actual odor but an odor plume like a weak song or light can still elicit a hedonic estimation. It also must be noted that hedonic scores show larger variations specifically for pleasant odorants (i.e., moderately pleasant), compared to unpleasant odorants.

Third, when hedonic scores were analyzed as a function of concentrations, i.e., independently of IDT, a general polynomial regression curve took shape with an increase of pleasantness/unpleasantness ratings. For pleasant odors, the higher the concentration, the more pleasant the odorant, and for unpleasant odors, the higher the concentration, the more unpleasant the odorant. These findings are in line with previous published works^13,35^. However, a large dispersion on either side of the curve was observed for all odorants. Thus, the relation noted between the odorant concentrations and the hedonic scores did not appear very informative at a specific concentration. In other words, the large dispersion of the hedonic scores observed at a specific concentration in a general population was probably due in large part to a methodological bias.

When the IDTs were considered, the general shape of the polynomial regression curve—although always positive—appeared differently oriented for all odorants except for trimethylamine. Specifically, with apple-jasmine and durian odors, the curve indicated a strong increase of pleasantness and unpleasantness ratings, respectively, which was followed by a plateau for the highest concentrations. With trimethylamine, the hedonic scores curve presented a plateau for the first concentrations above the threshold, followed by a strong increase of unpleasantness ratings for the highest concentrations.

Results also demonstrated that, when IDT was taken into account, the correlation coefficient was significantly higher for trimethylamine. A trend towards significance was observed for apple, while no difference was found for jasmine and durian. Other investigations based on a larger number of odors are needed to get a better understanding of this relationship, especially in relation to properties of odors (physicochemical properties, trigeminal activation, odor qualities e.g., food and non-food odors…).

The present findings are consistent with previous works^37–39^ suggesting such a relationship with a plateau. As trimethylamine activates more significantly the trigeminal system than durian, the involvement of trigeminal nerve activation could have an impact of the curve shape^55,56^. Indeed, the trigeminal system conveys sensory inputs especially related to irritation and pain. In the present study, results obtained with trimethylamine (i.e., a plateau following be a sharp increase of unpleasantness ratings) is linked to the Steven’s power law in which the curve representing the relationship between stimulus intensity and perceived intensity is similar to the one obtained in the present study. Besides, this result is consistent with those of Moskowitz et al.^35^ showing a similar relationship between higher concentrations and perceived unpleasantness using another trigeminal odorant (cyclohexanone). Further research in this field should thereby consider the trigeminal component of odorant stimuli, especially with higher concentrations.

In the present work, the effect of sex and hunger level was investigated but no significant results were found. Indeed, no difference in IDTs and OHRs was observed between men and women, knowing that the sex ratio was unbalanced in the sample. Because of the well-established sex differences in human odor sensitivity and odor hedonic perception^18,57^, this effect could be thoroughly examined in future research with a larger sample composed of a homogenous number of men and women. Likewise, no significant difference in hunger level was found between each odor session and participants hunger level had no influence on detection thresholds and OHRs at detection threshold and pure solution.

Some factors in the present study could be further investigated. First, three out of the four presented odor stimuli (i.e., durian, trimethylamine, and apple) corresponded to food related odors. In future research, it would be of interest to investigate the question of odor edibility as this is another relevant dimension in olfactory perception^58^ which could have an impact on odor pleasantness. Similarly, odor familiarity was not considered and warrants further exploration. Indeed, experience with odors constitutes a major factor modulating olfactory perception and previous studies have reported a relationship between ratings of familiarity of a given odor and ratings of pleasantness^59,60^. Moreover, no age effect was investigated in the present work insofar as all participants were students aged between 21 and 26. However, there is evidence that age can influence OHR^11,12^. Thus, it would be interesting to examine the age effect on OHR in relation with IDT. Finally, it would be interesting to conduct the same study with a larger sample size to confirm the present findings and to investigate the age effect. From a methodological point of view, it must be noted that the inter-stimulus interval was not exactly the same within subjects and between subjects, which could induce sensory fatigue or habituation. Moreover, considering that a session lasted between 25 and 40 minutes, a possible sensory fatigue or habituation could have affected the results and must be studied in future investigations.

Taken together, these findings suggest a potential relationship between odor hedonic ratings and individual sensitivity. It could be considered in studies in this field as other classical well-known parameters listed in the introduction section^61^. Interestingly, when IDTs were considered, the variation of hedonic ratings for concentrations above the threshold was consistent among subjects suggesting a strong intrinsic correlation between odor intensity and hedonic ratings. Thus, it is highly probable that this relation was masked in most of studies by scores flexibility due to a methodological bias. It would be relevant to consider this fact in future research. For instance, a lot of works focused on the role of physicochemical properties of odorants and perceptual characteristics, especially the hedonic perception^5,8^ and experiments could examine this link at the light of the present findings. On the other hand, a lot of works investigated the odor hedonic dysfunctionning in several psychiatric disorders (depression, schizophrenia...) and neurodegenerative diseases (Parkinson’s disease, Alzheimer disease...) and comparisons with control populations would undoubtedly offer more robustness to the results if individual sensitivity was also considered. Finally, insofar as the sensitivity for a subject can vary over time in relation to parameters such as age, physiological state, level of hunger, disease, medication... longitudinal studies could more closely explore the relationship between sensitivity and hedonic ratings. Overall, a better understanding of the relationship between sensitivity and hedonic ratings appears relevant because of the importance of odors perception in daily life and specifically the odor hedonic perception in relation to the quality of life.

## Data Availability

The datasets used and/or analyzed during the current study available from the corresponding author on reasonable request.
